# An Adaptive Two-Dimensional Voxel Terrain Mapping Method for Structured Environment

**DOI:** 10.3390/s23239523

**Published:** 2023-11-30

**Authors:** Hang Zhou, Peng Ping, Quan Shi, Hailong Chen

**Affiliations:** 1 School of Transportation and Civil Engineering, Nantong University, Nantong 226019, China; 2118320016@stmail.ntu.edu.cn (H.Z.); hilong521@163.com (H.C.); 2 School of Aeronautics and Astronautics, Chongqing University, Chongqing 400044, China

**Keywords:** terrain mapping, foot robot, plane extraction, preemptive RANSAC, adaptive voxel

## Abstract

Accurate terrain mapping information is very important for foot landing planning and motion control in foot robots. Therefore, a terrain mapping method suitable for an indoor structured environment is proposed in this paper. Firstly, by constructing a terrain mapping framework and adding the estimation of the robot’s pose, the algorithm converts the distance sensor measurement results into terrain height information and maps them into the voxel grid, and effectively reducing the influence of pose uncertainty in a robot system. Secondly, the height information mapped into the voxel grid is downsampled to reduce information redundancy. Finally, a preemptive random sample consistency (preemptive RANSAC) algorithm is used to divide the plane from the height information of the environment and merge the voxel grid in the extracted plane to realize the adaptive resolution 2D voxel terrain mapping (ARVTM) in the structured environment. Experiments show that the proposed mapping algorithm reduces the error of terrain mapping by 62.7% and increases the speed of terrain mapping by 25.1%. The algorithm can effectively identify and extract plane features in a structured environment, reducing the complexity of terrain mapping information, and improving the speed of terrain mapping.

## 1. Introduction

Due to the structural characteristics of foot robots, the utilization of terrain information is crucial for foot landing planning and motion control. Foot robots are mainly utilized in industrial settings and structured terrains, like tall buildings. Therefore, establishing precise terrain mapping in these environments is essential for enhancing the motion efficiency of foot robots [1].

For legged robots, perception sensors are crucial for estimating terrain during movement [2]. However, these sensors, such as depth cameras and Lidar sensors, often face challenges like occlusion, varying light conditions, and motion blur. Notably, prevalent foot robots like ANYmal [3] or the Oncilla robot [4] typically feature depth cameras solely in the head position, leading to blind spots in critical foot planning areas, such as below the robot. To address this, foot robots must construct a topographic map using terrain information obtained from perception sensors. This map necessitates continuous integration and updates over time to estimate the surrounding terrain [5]. Simultaneously, terrain mapping must provide parameters like height and depth in a structured terrain to the planning controller. This aids in selecting appropriate landing points and facilitating motion control for movement in unconventional terrains [6]. While methods like Elevation Mapping [7] or Voxblox [8] mitigate the impact of robot-positioning drift and sensor noise, they often rely on heuristic methods and parameters, especially for outdoor applications with complex terrains. Handling structured terrains indoors, however, poses additional challenges. Importantly, existing terrain mapping methods seldom address terrain parameter estimation in structured scenes, leading to a lack of detailed terrain parameters for the robot’s foot planning and motion controller.

To accurately and efficiently map indoor structured terrain and obtain terrain parameters, this paper introduces a novel adaptive two-dimensional voxel terrain mapping method. The impact of sensor noise and pose estimation uncertainty is mitigated through the mapping coordinate frame. Additionally, the random sampling consistency algorithm [9] is incorporated into the process of mapping height information, enhancing its applicability to structured terrain. Lastly, mapping efficiency is heightened by adjusting the adaptive resolution of the map. Our contributions are outlined as follows:

(1) Through the terrain mapping framework, the environmental point cloud undergoes transformation into height information, which is subsequently mapped onto a two-dimensional voxel grid. This height information is then downsampled to streamline the data structure and reduce redundancy.
(2) The utilization of the preemptive RANSAC algorithm for plane extraction from the terrain height information within the voxel grid enables the estimation of parameters such as height and depth in structured environments.
(3) To accommodate the characteristics of various planes, an adaptive resolution strategy is employed to adjust and merge the resolution of the voxel mesh within the extracted planes. The voxel meshes from different planes are then seamlessly integrated. A specialized data structure is utilized to efficiently store the final terrain mapping information.
(4) An environment awareness system is constructed based on the Unitree Go1 platform. This system is deployed on an NUC10 embedded computer, and terrain mapping tests and evaluations are conducted in both simulation and real-world scenarios.

The paper is organized as follows: Section 2 summarizes relevant terrain mapping methods and the current research progress in this direction. Section 3 provides a detailed overview of the implementation process of the terrain mapping method in this paper. In Section 4, the experimental results of the algorithm in the simulation and the real world are presented, and the algorithm’s performance is evaluated. Section 5 summarizes the current progress of the work and discusses future endeavors.

## 2. Related Work

Currently, the perception of the surrounding environment by mobile robots predominantly relies on depth cameras or Lidar sensors to model their environment. These sensors capture point cloud data from the environment, which, when combined with the robot’s pose information, is used to generate raster maps [10,11,12], elevation maps [13,14,15], or three-dimensional point cloud maps [16,17,18]. Generally, for legged mobile robots, landing point planning and motion control can be achieved using low-dimensional representations, eliminating the need for full three-dimensional space maps. For instance, Boston Dynamics’ Spot Mini requires landing point planning on a map with a 1 cm resolution based on the size of its foot sole. However, existing three-dimensional maps at this resolution typically operate at a speed of around 1 Hz [19].

Elevation maps designed for legged robots usually involve mapping the height of the environment to create a flat grid of sheets. Fankhauser and colleagues [7] proposed a similar method. They fused the ranging information from distance sensors with the robot’s position and attitude through a Kalman filter, resulting in an elevation map of the surrounding terrain based on probabilistic formulas. However, this method exhibits some ambiguity in edge processing, particularly for mapping structured terrains where accuracy falls short. Building upon this work, M. Stolzle et al. [20] extended it to some extent and used a neural network to fill gaps in the height map. However, they did not filter the data from the visible areas, which could lead to data filling in occluded areas and consequently affect the accuracy of terrain mapping. Yang. B et al. [21] introduced a real-time neural dense Elevation Mapping method with uncertainty estimation, suitable for mapping terrain from sparse and noisy point cloud data. Zhang et al. [22] proposed a method to extract line features using laser scanning points and construct peripheral maps through feature matching technology. While these methods perform well in simpler structured environments, they face significant challenges in complex terrains, such as stairwells, especially in identifying the structural plane of stairs.

In voxelized occupancy diagrams, a two-dimensional height map corresponds to a three-dimensional occupancy grid diagram. Each voxel is assigned a binary label to store information about space occupancy, and the occupancy information of each label is updated through a Bayesian filter. These mapping computations typically demand significant memory resources but can be optimized by employing efficient memory representation methods like octrees [23,24]. Additionally, other representations such as Euclidean Symbolic Distance Fields (ESDF) [25] and Truncated Symbolic Distance Fields (TSDF) [26,27,28] map the distance information from point clouds obtained by distance sensors to the nearest voxel grid.

In addressing the feature detection requirements for structured terrains like stairwells, existing research generally adopts methods to segment the stair plane from point cloud data, such as the RANSAC method for stair plane extraction [29]. In the study by Wu, Y et al. [30], RANSAC was used to segment and extract point clouds based on the semantic structure of pairwise orthogonal planes in indoor scenes. This method accurately and effectively extracts the interior plane structure. Yang, LN et al. [9] proposed an indoor plane detection method based on spatial decomposition and an optimized RANSAC algorithm to overcome the issues of traditional RANSAC, which can lead to plane extraction errors. Candidate points are selected through a heuristic search strategy, and the final plane is estimated using these candidate points. In the plane extraction method proposed by Su, ZH et al. [31], it was noted that the RANSAC algorithm can lead to excessive plane segmentation, which results in reduced accuracy in plane extraction. In cases where planners require topographic parameters to support foot robots in foot placement planning and motion control, Wu et al. [32] introduced the preemptive RANSAC method into the process of four-legged robot movement in stairwells. This approach estimated the height and depth of stairs by extracting the stair plane, providing support for machine movement planning.

In comparison to these methods, our approach utilizes the preemptive RANSAC algorithm to extract planes in structured terrains based on the terrain mapping framework. Additionally, we use an adaptive voxel mesh to adjust the grid resolution in recognized planes to splice voxel meshes with different resolutions, ultimately completing the terrain mapping process. This method not only eliminates robot positioning drift and sensor noise but also effectively identifies and extracts structured planes, acquires terrain parameters, and ensures the accuracy and consistency of terrain mapping.

## 3. Methods

**Figure 1:**
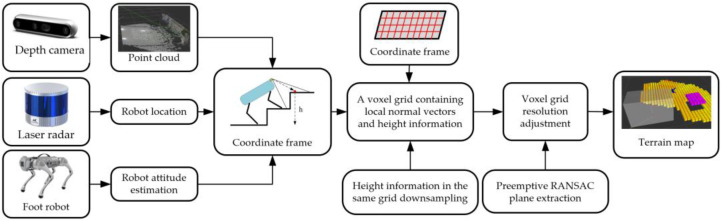
Algorithm general flow chart.

In this paper, the terrain mapping algorithm primarily encompasses the construction of a terrain mapping coordinate frame, the design of a two-dimensional voxel mapping grid, the extraction of planes from height values, and the subsequent adaptive resolution adjustment. The specific algorithmic flow is depicted in Figure 1 below:

### 3.1. Development of a Terrain Mapping System for Legged Robots

#### 3.1.1. Coordinate Framework for Terrain Mapping

**Figure 2:**
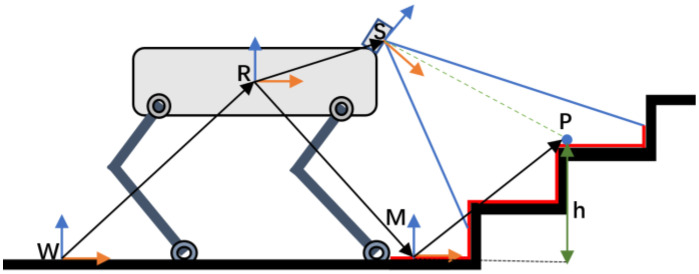
Terrain mapping coordinate system.

With the robot as the center, the entire terrain mapping system comprises four coordinate systems. As illustrated in Figure 2, these are the robot body coordinate system (R), the sensor coordinate system (S), the terrain mapping coordinate system (M), and the world coordinate system (W). The world coordinate system (W) remains stationary in the environment. Both the robot body coordinate system (R) and the sensor coordinate system (S) are also stationary, and there exists a known coordinate transformation $\left(r_{RS},\;\varphi_{RS}\right)$ between them. Leveraging the attitude information of the sensor and the robot body, we can derive terrain mapping for the surrounding point cloud concerning the world coordinate system. The terrain mapping coordinate system always maintains parallel alignment with the *Z* axis of the world coordinate system, while the azimuth of the X and Y axes may change.

Considering the link between the sensor, the robot body, and the environmental point cloud information collected by the distance sensor, we transform this information into a terrain mapping framework using the robot’s own position and orientation:

(1) $$p_{M}=p_{S}\cdot R_{S}^{M_{T}}\left(q\right)-t_{S}^{M}$$

here, $p_{S}$ and $p_{M}$ represent the vector representation of point P in the coordinate frames S and M, respectively. $R_{S}^{M_{T}}\left(q\right)$ represents the rotation matrix from frame S to M of the coordinate system, with the parameter *q* denoting the rotation angle between the two frames. $t_{S}^{M}$ represents the transformation from the sensor frame to the terrain mapping frame. Using this mapping framework, we can convert the sensor-measured point cloud information around the robot into height information values h for each point in the cloud. This can be expressed as Formula (2):

(2) $$h=P(\varphi_{SM}^{-1}\left(S^{r_{SM}}\right)-M^{r_{SM}})$$

#### 3.1.2. Construction of Two-Dimensional Voxel Grid for Topography Mapping

To process the height information within each terrain grid, we establish a two-dimensional voxel grid representation in which each voxel stores the height information of the terrain.

(1) Height information and position information of the point cloud can be mapped from each point cloud, and the side length of the voxel grid is set to L. Then, the two-dimensional voxel mesh can be subdivided into SUM=M×N daughter voxel meshes:

(3) $$\left\{\begin{array}{c} M=round[\left(X_{max}-X_{min}\right)/L]\\ N=round[\left(Y_{max}-Y_{min}\right)/L] \end{array}\right.$$

where the maximum value $X_{max}$ and $Y_{max}$ and the minimum value $X_{min}$ and $Y_{min}$ of the height information of terrain mapping in X and Y coordinate systems in two directions. round() is the integer function. Then, we subtract the minimum distance value from the maximum distance value to the side length $L_{x}$ and $L_{y}$ of the 2D terrain map.

(2) Encoding the two-dimensional voxel grid, the spatial index $\left(x_{i},\;y_{i}\right)$ of each voxel grid can be determined:
  (4) $$\left\{\begin{array}{c} x_{i}=round\left[\left(X_{i}-X_{min}\right)/L\right]\\ y_{i}=round\left[Y_{i}-Y_{min})/L\right] \end{array}\right.$$

(3) The terrain mapping height value obtained by point cloud transformation in each grid is traversed, the point cloud height mapping value in each grid is maximized, and the other point cloud height mapping values are removed.

(5) $$h_{i}=max\sum_{j=1}^{k}h_{j}$$

where k represents the number of point cloud mapping heights in the current voxel grid. After adding a spatial index to the height mapping values, a preliminary two-dimensional voxel terrain mapping system can be obtained as $p_{i}=(x_{i},y_{i},h_{i})$, where $x_{i}$ and $y_{i}$ are defined by the position of grid cell *i*, and $h_{i}$ is the estimated terrain height of cell i.

In the original two-dimensional voxel terrain mapping system, the local normal vector $\overset{⃑}{n_{i}}$ of the point cloud with height value is retained as the prime-centered normal vector of the corresponding voxel grid, which is used to extract the plane using the preemptive RANSAC algorithm.

### 3.2. Plane Segmentation from Height Values

In this paper, the preemptive RANSAC algorithm is used to extract the stair plane and estimate the stair parameters directly from the height information mapped into the voxel grid. The local normal vector and the height limit are added to the extraction process of the staircase plane to realize the extraction of a multi-level plane. The specific plane extraction process is as follows:

(1) The height values in the three-voxel grid are randomly selected from the voxel grid. If the three-voxel grid is not collinear, the corresponding plane $M_{i}$ is calculated.
(2) Calculate the difference between the height in the remaining grid and the height of the plane, respectively, $h_{d_{i}}$.
(3) An appropriate height threshold *d* is selected. If $h_{d_{i}}\leq d$, the voxel grid is divided into a voxel grid in the plane; otherwise, it is an out-of-plane voxel grid, and the number of voxel grids N in the plane is counted.
(4) Cycle the above three steps and iterate for K times. When *N* in this plane is the largest, it is identified as the best plane.

According to process Section 3.1, the measurement error satisfies the Gaussian distribution of ω standard variance and zero mean. The square $d^{2}$ of the difference between the height in the inner voxel grid and the height of the real terrain is the sum of the squares of the Gaussian variables, so it satisfies the $X_{v}^{2}$ distribution of degrees of freedom v. For example, when estimating the plane model, the difference between the height value in the voxel grid and the height of the plane is d; set d~N(0, ω), so $d^{2}\sim X_{1}^{2}$. In the following formula, the geometric distance from the data point (x, y, h) to the model is:

(6) $$d^{2}=d{\left(x,\hat{x}\right)}^{2}+d{\left(y,{\hat{y}}^{\prime }\right)}^{2}+d{\left(h,{\hat{h}}^{\prime }\right)}^{2}$$

Assuming that $d\left(x,\hat{x}\right)$, $d\left(y,\hat{y}\right)$, and $d\left(h,\hat{h}\right)$ all obey $N(0,\;\omega G_{3})$, and $G_{3}$ is the identity matrix of 3 × 3, we can obtain:

(7) $$d^{2}=d{\left(x,\hat{x}\right)}^{2}+d{\left(y,\hat{y}\right)}^{2}+d{\left(h,\hat{h}\right)}^{2}\sim X_{1}^{2}$$

When the probability of accepting the endovoxel grid is μ, the distance threshold can be set as:

(8) $$n^{2}=F_{V}^{-1}\left(\mu \right)\omega^{2}$$

As shown in Formula (9), the inner and outer voxel grid is divided into:

(9) $$\left\{\begin{array}{c} interior\;voxel\;d^{2}<n^{2}\\ outer\;voxel\;d^{2}\geq n^{2} \end{array}\right.$$

The number of iterations k of the algorithm can be inferred from the theoretical results. In the estimation of structured terrain, p is the probability that all the random grids selected from the voxel grid are internal voxel grids, and g is the probability that the in-plane voxel grid is selected from the voxel grid each time. It can be roughly estimated that the value of g in the structured terrain is the ratio of the number of in-plane grids to the total number of grids. Assuming that the estimated plane model requires the selection of n voxel meshes, $g^{n}$ is the probability that all n voxel meshes are inner voxel meshes, and $1-g^{n}$ is the probability that at least one of the n meshes is an outer sample mesh, indicating that the plane estimation model is poor. ${\left(1-g^{n}\right)}^{k}$ indicates that all N-voxel grids are inner grids, whose value is equal to  1−p. Logarithm of the n-voxel grids can be obtained as follows:

(10) $$k=\log\left(1-p\right)/log(1-g^{n})$$

The termination threshold *N* is a crucial parameter that dictates when the random sampling consistency algorithm should conclude. Typically, termination depends on the estimated value t of the internal voxel grid proportion g. When the number of voxel grids in the consistent data set reaches the predefined internal voxel grid standard, *N* can be set as N=tb, where b represents the total data size.

**Figure 3:**
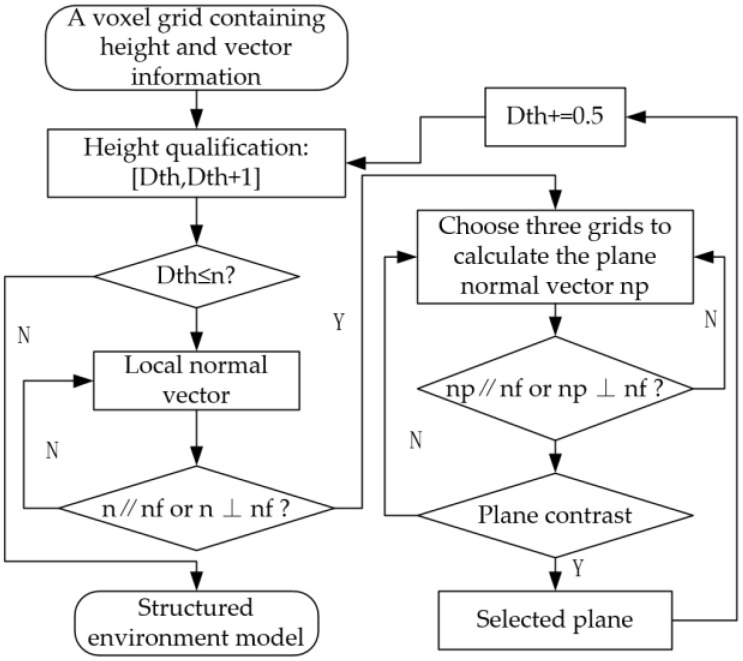
Flow chart of plane extraction algorithm.

To extract multilevel planes, we have introduced height and local normal vector constraints. The height constraint involves dividing height values in the voxel grid into height ranges, extracting planes within each range, and iteratively adjusting the height values. The local normal vector constraint uses the terrain mapping surface’s normal vector to determine whether the local normal vector of the point cloud corresponding to the height value in the voxel grid is parallel or perpendicular to the terrain mapping surface’s normal vector. If the result is otherwise, the voxel grid containing the height value is excluded. The specific algorithm flow is illustrated in Figure 3 below.

**Figure 4:**
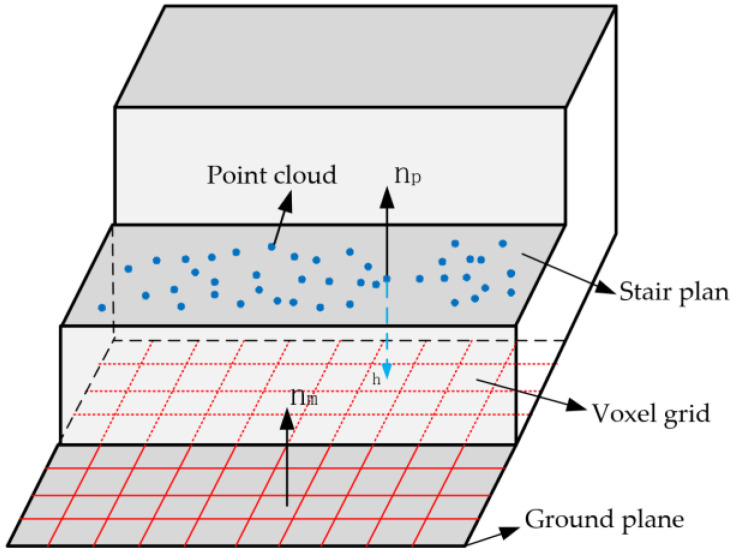
Structured terrain vector diagram.

The increasing value of the height limit can be adjusted according to the environmental conditions, thus avoiding missing detection. After the optimal plane is extracted, the Angle between the local height normal vector $\vec{n_{p}}$ of the voxel grid in the plane and the map normal vector $\vec{n_{m}}$ is determined to be $\theta_{pm}$, and the threshold of the Angle is set to $\theta_{tol}$,as shown in Figure 4 below.

Determine whether the included Angle $\theta_{pm}$ is less than the included Angle threshold $\theta_{tol}$. If yes, determine that the plane is a structured plane parallel to the map plane. The expression is shown below.

(11) $$\theta_{pm}=\arccos\left(\frac{\vec{n_{p}}\cdot \vec{n_{m}}}{\left|\vec{n_{p}}\right|\left|\vec{n_{m}}\right|}\right)\leq \theta_{tol}$$

In the preemptive RANSAC algorithm, plane extraction is mainly restricted by the conditions, and the goal is to extract the most suitable plane rather than the largest plane. The local normal vector restriction mainly excludes non-parallel and non-vertical planes from the ground plane to improve the accuracy and efficiency of plane extraction. Height restriction is mainly achieved by segmenting the height range in the voxel grid to achieve structured plane extraction, which can prevent the interference of environmental point clouds and other level planes, and make the staircase plane extraction more accurate.

### 3.3. Adaptive Voxel Grid Resolution

After extracting the plane in the structured environment, as described in Section 3.2, the height, depth, and other information about the plane in the structured environment can be obtained. Combined with the two-dimensional voxel terrain mapping system $p_{i}=(x_{i},y_{i},h_{i},\overset{⃑}{n_{i}},L_{x_{i}},L_{y_{i}})$ constructed in Section 3.1.2, we can master the position coordinates and specifications of the voxel grid contained in the plane. Thus, the voxel mesh contained in the extracted plane can be merged. Determine the voxel grid position of the edge in the plane that is closest to the original point on the map, and use it as the merging start position to merge the obtained voxel grid $L_{x_{i}}$ and $L_{y_{i}}$ in the plane. The following formula is shown:

(12) $$\left\{\begin{array}{c} L_{x_{i}}=\sum_{n=x_{i}}^{x_{imax}}L_{n}\\ L_{y_{i}}=\sum_{n=y_{i}}^{y_{i}max}L_{n} \end{array}\right.$$

$x_{imax}$ and $y_{imax}$ represent the maximum coordinate positions of the extracted voxel grids in the plane, and the voxel grids in the plane are merged into $p_{i}=(x_{i},y_{i},L_{x_{i}},L_{y_{i}},h_{i})$. $h_{i}$ is the height of the voxel grid, which is the plane. Finally, a two-dimensional voxel terrain mapping system with adaptive resolution is realized.

## 4. Experimental Validation

This section validates our approach through a series of experiments conducted on both simulated and real robots, in comparison with Elevation Mapping and Voxblox. The experiments primarily assess the performance of the proposed mapping algorithm from three dimensions: terrain fitting accuracy, terrain mapping error, and map updating time.

### 4.1. Simulation Experiment

#### 4.1.1. Construction of the Experimental Environment

**Figure 5:**
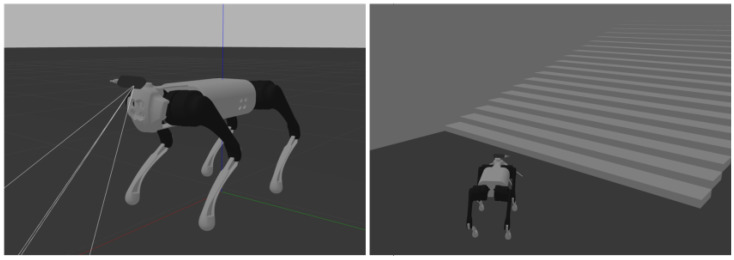
Simulation environment construction.

We simulated a structured environment in the Gazebo simulation environment, comprising elements like the ground plane, stairs, walls, and more. The parameters of these scene elements, such as the height and depth of the stairs, were adjustable to closely match real structured environments. The robot used in the simulation is based on the Unitree Go1 model, and it uses simulated Intel RealSense depth camera data, which aligns with our real-world experimental robot setup. The experimental environment is depicted in Figure 5 below:

#### 4.1.2. Simulated Experiment

Adhering to international building codes, the height and depth of the stairs in the simulation experiment were configured to be 15 cm and 32 cm, respectively. The point cloud data in the simulated environment was derived from the camera’s point cloud information which was collected within one meter in front of the robot. During the experiment, the robot’s motion control and terrain mapping algorithm nodes were initiated separately. In the Gazebo simulation environment, the robot model’s motion was controlled using keyboard control nodes, allowing real-time retrieval of terrain mapping results.

**Figure 6:**
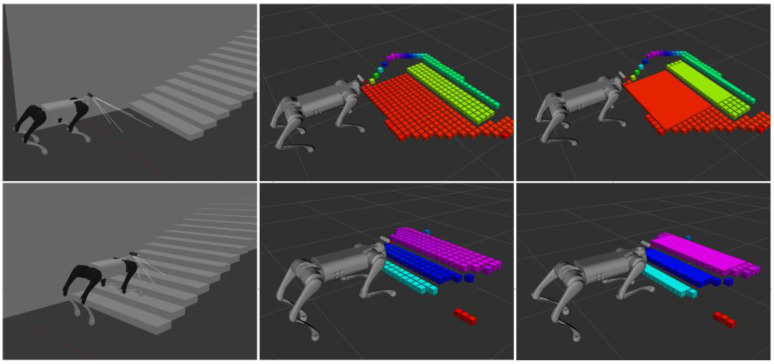
Terrain mapping simulation experiment.

Figure 6, presented above, displays the simulation outcomes of terrain mapping algorithms in various structured environments. In the simulated staircase environment, the robot ascends the stairs from the ground, evaluating the robot’s terrain mapping and plane extraction capabilities for both the ground and the staircase scene. Owing to the depth camera’s noise and the uncertainty in the robot’s position and attitude estimation, mapping errors may arise during edge estimation of the terrain. However, by constraining the terrain mapping range and extracting plane features, anomalous mapping points at the edges can be excluded, thus enabling precise mapping of the structured terrain.

### 4.2. Comparative Verification

#### 4.2.1. Experiment for Comparing Terrain Fitting Effects

**Figure 7:**
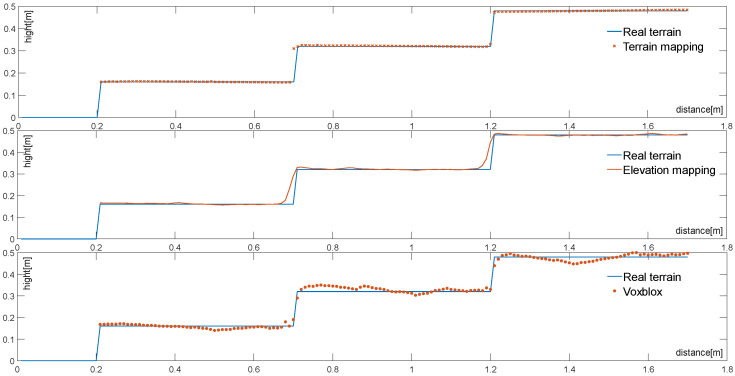
Cross-section of terrain mapping results.

Figure 7, depicted above, illustrates the terrain fitting map generated with different terrain mapping algorithms. In this figure, the *x*-axis and *y*-axis denote the direction and height of the terrain, respectively. These three mapping algorithms were evaluated using the same map resolution of 0.001 m. As observed in Figure 7, when confronted with the same structured terrain, Elevation Mapping and Voxblox exhibit slight deviations at the step’s edge. This behavior is primarily attributed to stronger sensor measurement errors near the step’s edge, causing fluctuations in plane terrain fitting due to inaccuracies in robot pose estimation. This results in an uneven map. However, with the incorporation of the preemptive random sampling consensus algorithm, the step’s plane is extracted independently. This separation diminishes the impact of sensor noise on measurements and leads to smoother and more accurate plane mapping.

#### 4.2.2. Comparison of Terrain Mapping Errors

To quantitatively compare the accuracy of various terrain mapping algorithms, this paper introduces a mapping error formula. This formula assesses mapping accuracy by contrasting the height value in the mapped voxel grid with the deviation in terrain height data from the environmental model. Let us assume there are n mapped voxel grids, each containing a height value $h_{i}$, and a corresponding true height value $h_{r}$. The error evaluation is presented in Formula (13) below:

(13) $$MapError=\sqrt{\frac{1}{n}\sum_{i=1}^{n}{\left(h_{i}-h_{r}\right)}^{2}}$$

**Figure 8:**
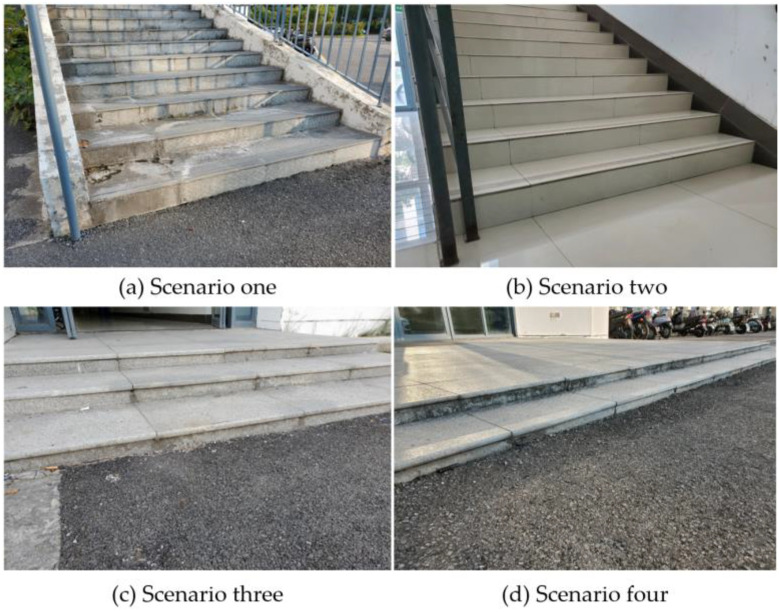
Four distinct structured environments.

To evaluate the algorithm’s mapping capabilities in various structured terrains, the experiment involved conducting error comparison experiments in different stair-like environments. As depicted in Figure 8 below, four representative stair environments with varying heights, depths, and numbers of steps were selected for the experiment. Simultaneously, mapping error tests were conducted under different map resolutions to assess how terrain mapping errors change with varying resolutions, specifically at map resolutions of 0.005 m, 0.01 m, 0.02 m, 0.05 m, and 0.1 m.

**Figure 9:**
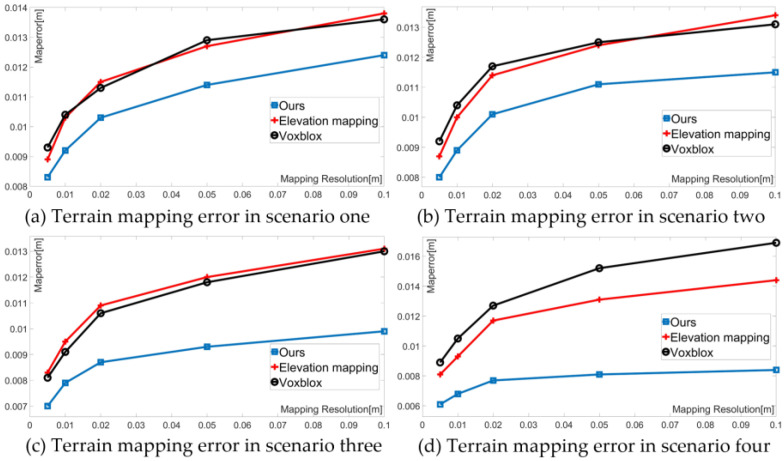
Terrain mapping error under different structured terrains and map resolutions.

The experimental results shown in Figure 9, corresponding to the four structured environments illustrated in Figure 8, clearly reveal the terrain mapping errors for the three terrain mapping algorithms across different structured terrains. As seen in Figure 9, under the same terrain resolution, the mapping error decreases with a reduction in step span and an increase in step depth. Additionally, as the proportion of planes increases, the errors in the Elevation mapping and Voxblox algorithms, which were comparable in (a), (b), and (c), become distinct in (d), where the error in Elevation mapping is smaller than Voxblox. Furthermore, with a decrease in map resolution, this trend becomes more pronounced. This improvement is primarily attributed to the incorporation of the terrain mapping framework, enhancing the accuracy of height estimation. In comparison with Elevation Mapping and Voxblox, the proposed algorithm exhibits the smallest terrain mapping error among the four structured terrain mapping methods. This indicates that the algorithm achieves a higher precision and better adaptability in structured terrains. Moreover, within the same structured terrain, the proposed algorithm demonstrates the smallest increase in the terrain mapping error with a reduction in map resolution compared to the other two methods. The introduction of plane extraction steps and adaptive resolution enhances the adaptability of the terrain mapping algorithm to structured terrains.

#### 4.2.3. Comparison of Terrain Mapping Speed

To quantitatively compare the terrain mapping speed of three different terrain mapping algorithms, we conducted an experiment using the same structured terrain point cloud data while monitoring the robot’s pose changes. The environmental data collected pertained to a structured stairwell with dimensions of 5 m in length and 3 m in width. Each of the three terrain mapping algorithms was employed to construct a complete map.

The calculation times for maps with different resolutions in the same environment are presented in Table 1 below. When the algorithm transforms the point cloud into height information and maps it to the voxel grid, the height information within the same grid is downsampled, resulting in a significant reduction in the height information processed by the algorithm. Consequently, the computational workload is decreased when mapping the same point on the same terrain. This reduction leads to a corresponding decrease in the algorithm’s processing time.

### 4.3. Real Terrain Testing

**Figure 10:**
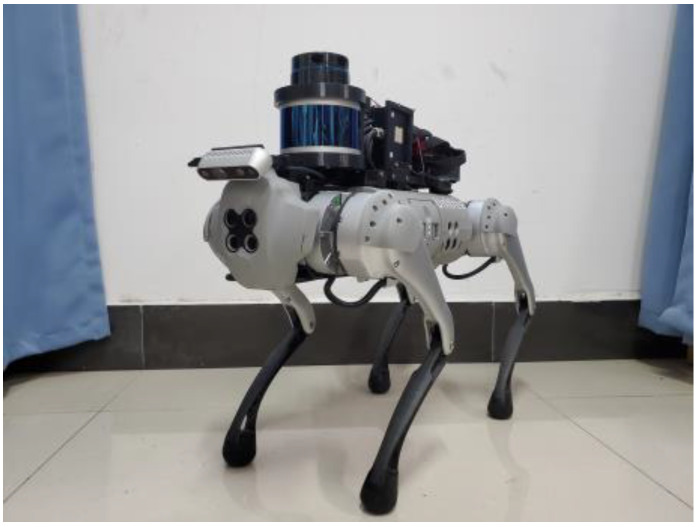
Hardware diagram of sensing system for quadruped robot.

The real-world experiments were conducted using a custom-built four-legged robotic system equipped with a depth camera and a Lidar sensor. The robotic platform used was a Unitree GO1 EDU quadruped robot, featuring 12 servo motor joints. The rear of the robot was outfitted with a 16-line laser radar and an IMU attitude sensor for precise position and attitude estimation. The front of the robot was equipped with an Intel D435 depth camera, which captures point cloud data from the environment for distance measurements. The overall hardware configuration is depicted in Figure 10. The terrain mapping and motion control algorithm were implemented on an Intel NUC10 located on the robot’s back. This computing unit is equipped with an Intel Core i7 10700k processor and 16 GB of memory. The entire algorithm was developed in C++ and includes integration with the Robot Operating System (ROS). The mapping is configured to cover distances within one meter from the robot’s center.

The experiments involved comparing the performance of various terrain mapping methods in the same scene initially. Subsequently, a more representative scene, encompassing ground, stairs, and other structured terrains, was chosen to evaluate the effectiveness of the algorithm proposed in this paper. Furthermore, a preliminary experiment was conducted in a semi-outdoor setting.

**Figure 11:**
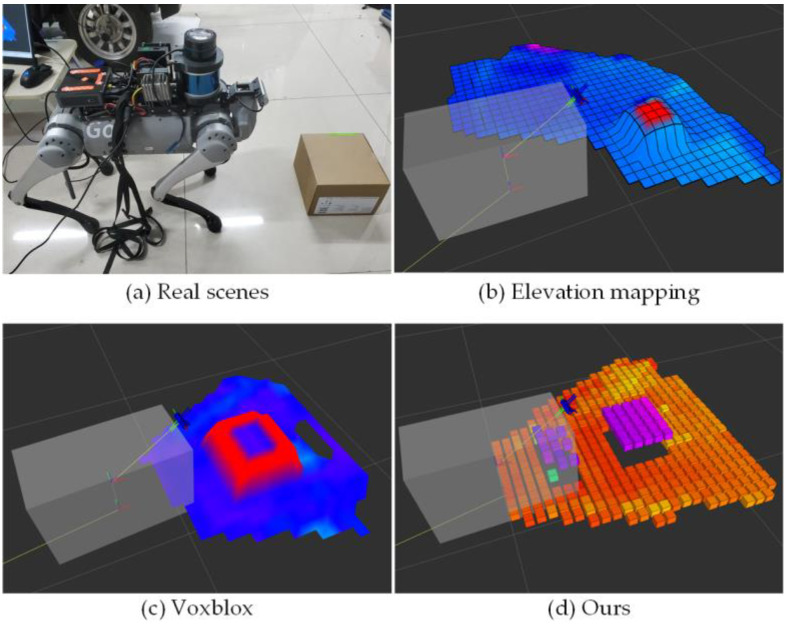
Comparison of three mapping effects under the same terrain.

Figure 11 below provides a comparative analysis of the effects of three terrain mapping methods in the same terrain scene. Here, (b), (c), and (d), respectively, represent the terrain mapping algorithm presented in this paper, Elevation Mapping, and Voxblox. As depicted in Figure 10, it is evident that the terrain mapping method proposed in this paper, with the inclusion of the plane extraction step, yields sharper terrain height transitions and smoother plane mapping when compared to Elevation Mapping and Voxblox. During the plane extraction process, our input object is the height value rather than the point cloud. Each voxel grid contains only one height value, derived from the point cloud’s height value within the grid through the mapping framework and downsampling. This significantly reduces our computational data processing. In comparison with other terrain mapping methods, this approach can significantly decrease space complexity. Although our experiment was conducted on the NUC10, it did not fully utilize all of its computing resources. Additionally, we deployed and tested our algorithm on the NVIDIA NX. At a map resolution of 0.01 m, it can maintain a map update speed of approximately 8 Hz. In contrast, the other two terrain mapping methods can only sustain a map update speed of about 3–4 Hz.

**Figure 12:**
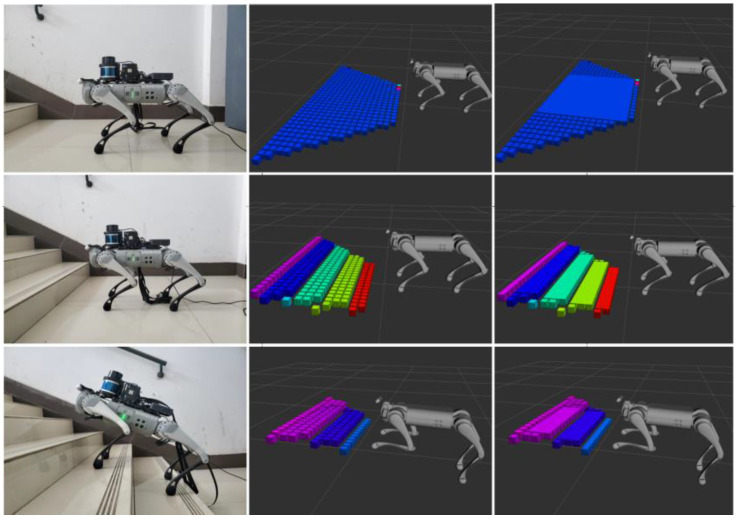
Indoor structured terrain mapping.

Figure 12 illustrates the algorithm’s performance across different structured terrains. In an indoor ground environment, the terrain mapping algorithm presented in this paper performs well, offering superior ground recognition and achieving high flatness. However, when it comes to recognizing a structured terrain like stairs, differences from traditional terrain mapping algorithms become apparent. Notably, the recognition of step edges can be slightly distorted due to noise present at the edges of the stairs. To address these issues, this algorithm adopts a preprocessing step where point cloud height information is mapped to the voxel grid, and heights within the same grid are filtered. This filtration effectively reduces the influence of sensor noise and the uncertainties stemming from robot pose estimation. Furthermore, the inclusion of the preemptive random sampling consistency algorithm enhances stair plane extraction, significantly improving terrain mapping accuracy.

**Figure 13:**
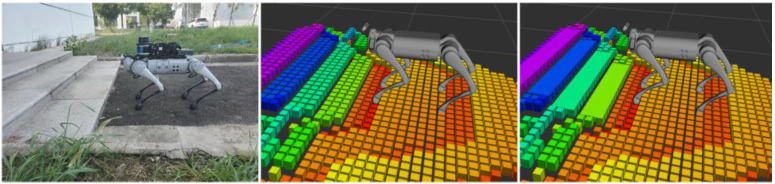
Semi-outdoor scene test.

Furthermore, due to the inclusion of the plane extraction process, the algorithm’s capacity for recognizing terrains in outdoor sports scenes remains somewhat limited. As demonstrated in Figure 13, within semi-outdoor environments, the algorithm can still effectively extract regular ground steps and other terrain information. However, for areas with nearby weeds and similar environments, terrain mapping proves to be challenging, yielding results with significant noise.

## 5. Conclusions

In this paper, we propose an efficient adaptive two-dimensional voxel terrain mapping for structured environments, which includes three key parts. Specifically, in the extraction process of environmental terrain height information, considering the impact of distance sensor noise and robot pose estimation uncertainty on height information estimation, the height information mapped to the voxel grid is downsampled to simplify the data structure and reduce the redundancy of information. This allows the robotic system to obtain the height information of the terrain more accurately, and effectively reduces the complexity of the data. Through plane extraction of terrain height information in a voxel grid using preemptive RANSAC, the estimation of height and depth of structured environment is realized. The resolution of the voxel mesh in the extracted plane is adjusted and merged by adopting the strategy of adaptive resolution, and the voxel mesh in different planes is spliced. To efficiently store the final terrain mapping information, custom data structures are also used. In this way, the details of different planes can be flexibly processed, and the information of the overall terrain can be effectively preserved. At the same time, the algorithm presented in this paper also demonstrates its limitations in unstructured terrain, which will be further addressed in subsequent research.

## Figures and Tables

**Table 1:** Terrain mapping speed at different resolutions.

Map Resolution(m)	Voxblox	Elevation Mapping	Ours
0.005	27.84 s	26.71 s	24.69 s
0.01	22.27 s	21.36 s	19.17 s
0.02	14.79 s	14.16 s	11.44 s
0.05	10.27 s	9.82 s	6.73 s
0.1	4.92 s	4.76 s	2.49 s

## Data Availability

Due to the privacy of the follow-up study, the data in this paper is unavailable at this time.
